# Band-offsets scaling of low-index Ge/native-oxide heterostructures

**DOI:** 10.1038/s41598-024-55851-7

**Published:** 2024-03-05

**Authors:** Bin Leong Ong, Eng Soon Tok

**Affiliations:** Electronic Materials Growth and Interface Characterisation (ƐMaGIC) Lab, Department of Physics, National University of Singapore, 2 Science Drive 3, Singapore, 117551 Singapore

**Keywords:** Surfaces, interfaces and thin films, Electronic properties and materials

## Abstract

We investigate, through XPS and AFM, the pseudo layer-by-layer growth of Ge native oxide across Ge(001), (110) and (111) surfaces in ambient environment. More significantly, our study reveals a universal set of valence and conduction band offset (VBO and CBO) values observed for Ge(001), Ge(110), and Ge(111) surfaces as a function of Ge-oxide concentration. We find that the band offsets appear to be the same across these low-index Ge surfaces *i.e.,* for Ge-oxide/Ge heterostructures with the same Ge-oxide overlayer concentration or thickness. In contrast, different oxidation rates for Ge(001), Ge(110), and Ge(111) surfaces were observed, where the oxidation rate is fastest for Ge(001), compared to Ge(110) and Ge(111). This can be attributed to the different number of unsatisfied Ge dangling bonds (2 vs 1) associated to the respective ideal Ge surface in forming Ge-oxide. Thus, at any given oxidation time, the oxide concentration or thickness for each type of low index Ge surface will be different. This in turn will lead to different band offset value observed for each type of Ge surface. More significantly, we show that while oxidation rates can differ from different Ge surface-types, the band offset values *can* be estimated simply based on the Ge-oxide concentration regardless of Ge surface type.

## Introduction

Ge, and Ge-related alloys such as SiGe and GeSn, are becoming major key players in the finFET and Gate-All-Around (GAA) FET industries^1–8^ due primarily to their higher attainable electron and hole mobilities relative to Si^9^. The integration of Ge as the main channel material in the form of single crystal nanowires and nanosheets for finFET^10^ and GAA-based devices^1,5,6,11^ will inevitably expose low-index surfaces such as (001), (110) and (111) at the interfaces between the channel, dielectric (oxide) gates, and source/drains contacts, given their relatively low surface energies. On another hand, device performance utilising these nanostructures are significantly affected by the crystal orientation of Ge. For instance, the effective hole mobilities of a < 110 > / < 110 > GOI pMOSFET is reported to be 2.3 times higher compared to (100)-oriented GOI control devices^12^.

Oxidation occurring along these low-index Ge-surfaces/oxide heterojunction interfaces can result in band-bending leading to the formation of band offsets with valence band-offset (VBO) values as high as 4.59 ± 0.03 eV^13–15^. The formation of the band offset at the Ge-oxide/Ge heterojunction arises due to charge transfer from Ge to the oxide layer, attributed to the presence of unreacted Ge ‘dangling’ bonds where charged carrier-trapped states may occur due to the presence of Ge with varying degree of unreacted ‘dangling’ bonds in the Ge-oxide matrix^14,16–18^. It is reported that these probable localized states can impact band offsets at semiconductor/insulator interface^18^ with theoretical values for VBO and CBO up to 3.98 ± 0.16 eV and 1.10 ± 0.16 eV respectively for Ge(100)/a-GeO_2_.

Previously, a universal band offset study was reported for Ge-oxide/Ge(001) heterojunction where an upward band bending of 0.6 eV is observed depending only on the amount of Ge oxide concentration^19^. Nonetheless, the Ge-oxide/Ge interface heterojunction interface is expected to differ when different low-index Ge surface is exposed, since the number of dangling bonds per unit area for each Ge surface type is expected to be different^20^. This difference, in turn, may also affect the band offsets, thereby affecting the performance of electronic devices employing Ge as the main channel materials for finFET and GAAFET devices. Furthermore, the oxidation reaction and oxidation rate of Ge from each low-index surface may also differ since the number of Ge-bonds per unit area for each low-index Ge surface is different. It is therefore critical to study and understand how the oxidation of other low-index Ge surfaces, namely Ge(110) and Ge(111), influence the offset values compared to Ge(001). In this work, we study the effects of oxidation from different low-index Ge surfaces on the band offsets of Ge-oxide/Ge heterojunction interface, as a function of exposure time over 500 days. We also address the differences arising from the different band offset values reported for different Ge-oxide/Ge by demonstrating that the offset values depend only on the extent of the oxidation of Ge, regardless of the Ge surface orientation.

## Results and discussion

**Figure 1:**
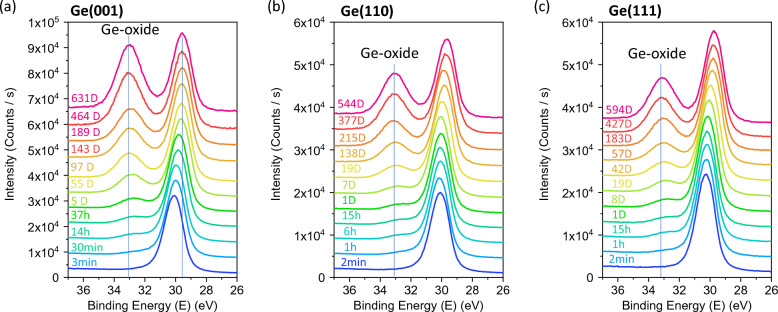
XPS Ge3d Spectra of (**a**) Ge(001), (**b**) Ge(110) and (**c**) Ge(111) plotted as a function of oxidation time (mins).

Figure 1 shows the Ge3d core-level spectra for (a) Ge(001), (b) Ge(110) and (c) Ge(111) surfaces. The initial oxidation of all three Ge surfaces forms a sub-oxide Ge-oxide (GeO_x_ and x < 2). This is attributed to the breaking of Ge–Ge bonds in the unoxidized Ge to form Ge–O bonds, thus leading to an increase in the oxidation states of Ge forming Ge^1+^ and Ge^2+^. As oxidation proceeds, more Ge–Ge bonds are broken to form Ge–O resulting in the formation of GeO_2_-like oxides, GeO_x_ (where x ≈ 2), containing Ge^3+^ and Ge^4+^. In all three substrate surfaces, the Ge3d^0^ doublet-peak for as-etched surfaces started with a binding energy (BE) of 30.0 eV, with no detectable Ge-oxides. The formation of the Ge-oxides started as early as 30 min, with the small shoulder peak at about 33.0 eV. The oxide peak increases in intensity with increasing exposure time to air (up to and over 500 days) while its peak BE increases only slightly from 33.0 eV to 33.1 eV, due to the initial formation of Ge sub-oxide which is then followed by more GeO_2_-like oxides. On the other hand, the substrate (elemental) Ge3d^0^ peak is observed to decrease with increasing exposure time, starting from 30.0 eV to about 29.5 eV. On closer examination, the oxide peak of Ge(001) is found to increase noticeably faster compared to Ge(110) and Ge(111) surfaces. At exposure times after 300 days, the peak intensity of Ge-oxide from Ge(001) is almost reaching the intensity of the substrate Ge3d^0^ peak intensity, while the oxide peak intensities from Ge(110) and Ge(111) surfaces remain relatively low at about half of their respective Ge3d^0^ peak intensities.

**Figure 2:**
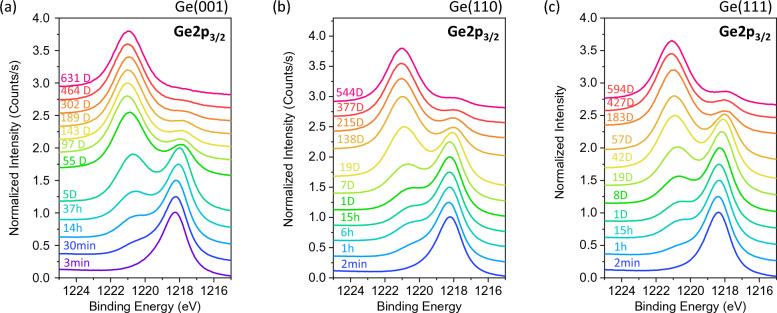
XPS Ge2p_3/2_ Spectra of (**a**) Ge(001), (**b**) Ge(110) and (**c**) Ge(111) plotted as a function of oxidation time (mins).

Figure 2 shows the Ge2p_3/2_ core-level spectra corresponding to the same set of Ge surfaces in Fig. 1. In this figure, the Ge2p_3/2_ BE of the substrate started at about 1218.4 eV but decreases with increasing time towards 1218.0 eV. The oxide peaks of the Ge-surfaces were minimal at 2 to 3 min of exposure to air, but they increase in intensity and BE from 1220.5 eV towards 1221.0 eV with increasing time. Similar to the Ge3d spectra in Fig. 1, the oxide peak from Ge(001) surface also appears to increase relatively faster compared to Ge(110) and Ge(111) surfaces. Above 400 days, the Ge2p_3/2_^0^ substrate peak is almost undetectable in Ge(001) while those from Ge(110) and Ge(111) surfaces still show a visible small peak.

**Figure 3:**
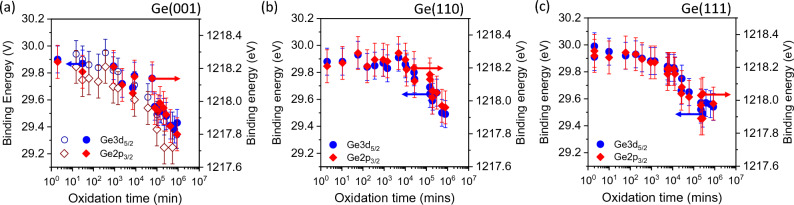
Ge3d_5/2_ and Ge2p_3/2_ binding energies plotted as a function of oxidation time for (**a**) Ge(001), (**b**) Ge(110) and (**c**) Ge(111). The decrease in the BE values with oxidation time for the three Ge substrates appear to be similar. In (**a**), Ge3d_5/2_ and Ge2p_3/2_ BE (blue and red empty points) are extracted from Ong et al. Appl. Surf. Sci. 530 (2020) 147–256.

The decrease in BE for both Ge3d and Ge2p_3/2_ core-levels of the substrate with increasing exposure time to air is shown more clearly in Fig. 3. In this figure, the peak BE of all three Ge surfaces started at about 30.0 eV in the initial exposure. The BE does not appear to change significantly up to 10^3^–10^4^ min (1 day–1 week) of exposure time. Above 10^4^ min, the BE decreases gradually with increasing time by as much as 0.6 eV towards 29.5 eV. In all three substrate surfaces, the decrease in BE is similar for Ge3d and Ge2p_3/2_. The changes in BE are also comparable to a previous work reported by Ong et al.^19^ using monochromatic X-Ray Al source. This decrease in BE is reported to be because of band-bending resulting from the transfer of charge from substrate to the oxide as the oxide grows with exposure time to air.

**Figure 4:**
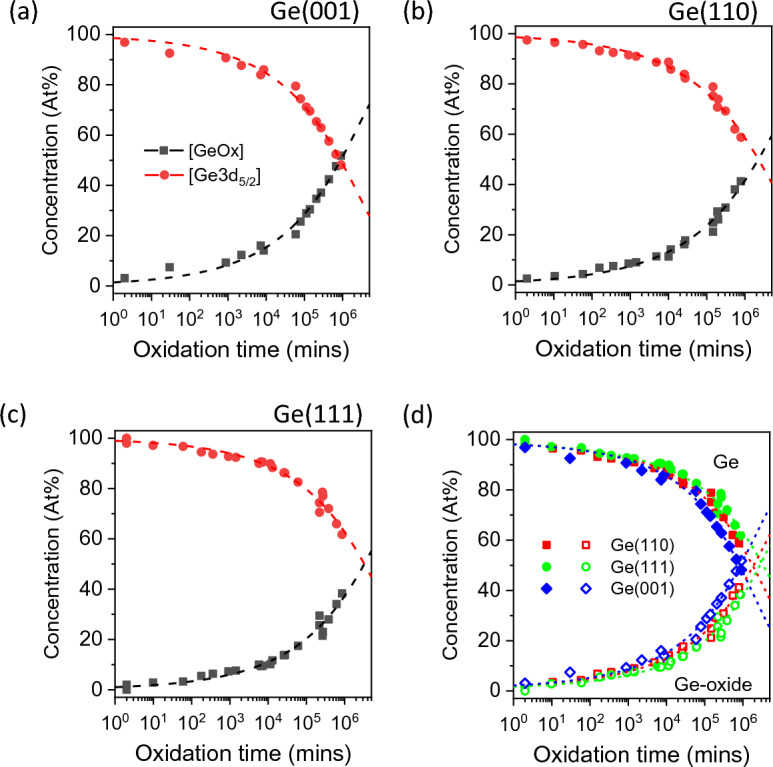
Ge and Ge-oxide compositions plotted as a function of oxidation time (log-scale) for (**a**) Ge(001), (**b**) Ge(110) and (**c**) Ge(111). In (**d**), the composition plots are combined to show the relative oxidation rates of the three Ge substrates, with Ge(001) being the fastest.

The growth of Ge-oxide due to exposure to air can be further quantified by comparing the concentrations of Ge-oxides to their substrate counterparts, as shown in Fig. 4. In this figure, the relative concentrations of Ge (${C}_{Ge3d5/2}\left(\text{at.\%}\right))$ and Ge-oxide (${C}_{Ge-oxide}\left(\text{at.\%}\right))$ are first quantified based on the Ge3d spectra from Fig. 1, i.e.

1 $${C}_{Ge3d5/2}\left(\text{at.\%}\right)=\frac{{I}_{Ge3d5/2}}{{I}_{Ge3d5/2} +{ I}_{Ge-oxide}}\times 100$$

2 $${C}_{Ge-oxide}\left(\text{at.\%}\right)=\frac{{I}_{Ge-oxide}}{{I}_{Ge3d5/2} +{ I}_{Ge-oxide}}\times 100$$

where ${I}_{Ge3d5/2}$ and ${I}_{Ge-oxide}$ are the normalized peak areas of Ge3d_5/2_ and Ge3d (oxide) peaks respectively. In Fig. 4a, the concentration of Ge-oxide relative to the unoxidized Ge-substrate increases steadily upon exposure to air up to 10^4^ min. The rate of oxide-growth then appears to change as a function of log-time, where it reaches about 50 at% Ge-oxide concentration at about 10^6^ min. Comparing to Ge(001) surface, Ge(110) and Ge(111) surfaces (Fig. 4b,c) appears to show similar oxidation behaviour. However, the increase of the oxide formation in the initial exposure to air seems to be slower for Ge(111) and Ge(110) compared to Ge(001). The slower rates of oxidation for Ge(111) and Ge(110) surfaces become more apparent when the three surfaces are plotted together as shown in Fig. 4d. As shown in this figure, it takes about 10^6^ min for Ge(001) to reach 50 at% Ge-oxide, while it takes over 10^6^ min for both Ge(111) and Ge(110) surfaces to reach 50 at% Ge-oxide. Thus, the increase in oxidation appears to be the slowest for Ge(111), followed closely by Ge(110) and then Ge(001) which has the fastest oxidation rate.

To further examine the increase in the oxide concentration as a function of time, the Ge-oxide overlayer is assumed to form on the Ge substrate (Ge) in a pseudo layer-by-layer mode. At photoelectrons’ emission angle normal to the sample surface, both the layer (Ge-oxide) and substrate (Ge) core-level intensities, ${I}_{Ge-oxide}$ and ${I}_{Ge3d5/2}$, can be then described by the following equations^21^:

3 $${I}_{Ge3d5/2}={I}_{Ge3d5/2}^{\infty }\mathit{exp}\left(-\frac{d}{{\lambda }_{Ge3d5/2}}\right)$$

4 $${I}_{Ge-oxide}={I}_{Ge-oxide}^{\infty }\left[1-\mathit{exp}\left(-\frac{d}{{\lambda }_{Ge-oxide}}\right)\right]$$

where ${I}_{Ge3d5/2}^{\infty }$ and ${I}_{Ge-oxide}^{\infty }$ are the core-level intensities of clean (unoxidized) and fully oxidized Ge(001) samples respectively; λ is the inelastic mean free path (IMFP) of the photoelectrons which can be estimated as λ ≈ 0.103 (KE)^0.745^^22^. For photoelectrons emitted from Ge3d_5/2_ and Ge-oxide (Ge3d) core-levels, the values of λ_Ge3d5/2_ and λ_Ge-oxide_ are 2.342 nm and 2.338 nm respectively; *d* is the oxide thickness in nm. Other assumptions for adopting this growth-model is that (i) the sample is flat and uniform, and (ii) the Ge-oxide/Ge interface is abrupt. The increase in oxide thickness *d* due to exposure to air can be empirically fitted using a simple power law^19^, where oxide thickness

5 $${d \left({\text{nm}}\right)=A\cdot t}^{n}$$

where *t* is the oxidation time in mins, and *A* and *n* are constants. The oxidation behavior is akin to the logarithmic law oxidation mechanism as described by Mott and Cabrera^23,24^ where the rate limiting step is related to the formation of GeO_2_ at the Ge/Ge-oxide interface, mediated by oxygen diffusing through the oxide layer where oxidation occurs at the interface. The oxidation proceeds such that Ge^n+^ (where n increases from 1 to 4) in a pseudo layer by layer mode.

By combining Eqs. (1) to (5), the relative concentrations of Ge and Ge-oxide can then be expressed as

6 $$\left[{\text{Ge3d}_{\text{5/2}}}\right]\left({\text{t}}\right)=\frac{{\text{exp}}\left(-\frac{A{t}^{n}}{{\lambda }_{\text{Ge3d5/2}}}\right)}{{\text{exp}}\left(-\frac{A{t}^{n}}{{\lambda }_{\text{Ge3d5/2}}}\right){+}\frac{1}{{\text{K}}}{\times}\left[{1}-{\text{exp}}\left(-\frac{A{t}^{n}}{{\lambda }_{\text{Ge-oxide}}}\right)\right]}{\times}{100\%}$$

7 $$\left[{\text{GeOx}}\right]\left({\text{t}}\right)=\frac{\left[{1}-{\text{exp}}\left(-\frac{A{t}^{n}}{{\lambda }_{\text{Ge-oxide}}}\right)\right]}{{\text{K}}\times {\text{exp}}\left(-\frac{A{t}^{n}}{{\lambda }_{\text{Ge3d5/2}}}\right){+}\left[{1}-{\text{exp}}\left(-\frac{A{t}^{n}}{{\lambda }_{\text{Ge-oxide}}}\right)\right]}{\times}{100\%}$$

where $K=\frac{{I}_{Ge3d5/2}^{0}}{{I}_{Ge-oxide}^{\infty }}$ is a constant. By using Eqs. (6) and (7) to fit the plot for all three Ge surfaces, the values of *A* and *n* can therefore be extracted as shown in Table 1a.

**Table 1:** Fitting parameters *n* and *A* extracted using (a) Eqs. (6) and (7) for Fig. 4, and (b) Figure Eq. (5) for Fig. 9.

Substrate	n	A (nm/min^0.25^)
(a)		
Ge(110)	0.25	0.096 ± 0.009
Ge(111)		0.084 ± 0.008
Ge(001)		0.116 ± 0.010
(b)		
Ge(110)	0.25 ± 0.01	0.095 ± 0.008
Ge(111)		0.083 ± 0.007
Ge(001)		0.115 ± 0.010

**Figure 5:**
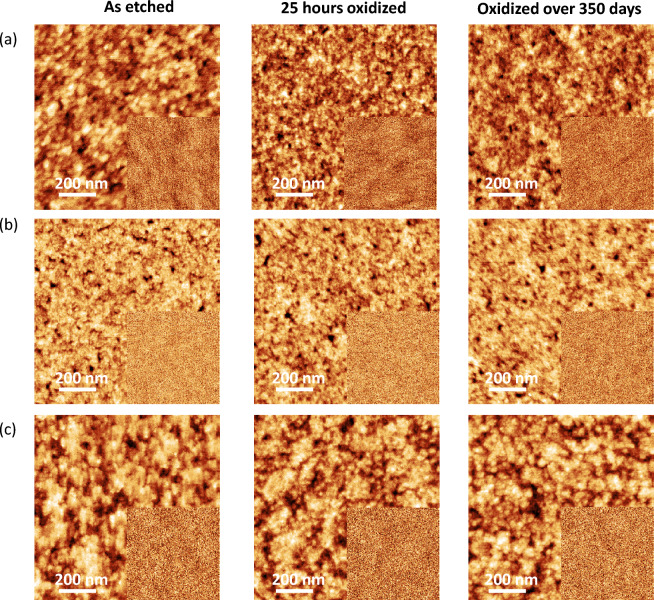
(**a**) Ge(001), (**b**) (110) and (**c**) (111) 1μm × 1μm surface morphologies obtained as etched, after 24h and after 350 days of oxidation. The surface roughness of the substrate surfaces did not exceed 0.51 nm after 350 days of oxidation in air. Inset: 10 μm × 10 μm scan sizes of the same substrate surfaces.

To assess the uniformity of the Ge-oxide layers, we then examine the surface morphologies of three Ge surfaces using AFM as shown in Fig. 5. The surface morphologies are taken immediately after deionized H_2_O etching, followed by after 25 h and 350 days of oxidation in air. The surface morphologies are similar for all three Ge surfaces, with no distinct features observed. Additionally, the surface roughness of each Ge surface does not exceed 0.51 nm even after 350 days of oxidation in air, suggesting that the Ge-oxide overlayer is uniform without 3-dimensional (Volmer-Weber) growth. The AFM results therefore suggest that the growth of the oxide layer is likely to be pseudo layer-by-layer (Frank van-der Merwe like) growth^25^. These results support our analyses of Ge-oxide growth using XPS.

The shifts in the BE of Ge3d and Ge2p_3/2_ core-levels show a similar trend in the decrease of the BE with increasing oxidation time for all Ge surfaces. However, with a slightly faster oxidation rate observed for Ge(001) compared to Ge(110) and Ge(111), it would be interesting to investigate how the band offsets in Ge-oxide/Ge(001) changes in comparison to that of Ge(110) and Ge(111). Band offsets, namely the valence band offset (VBO) and conducting band offset (CBO), can be extracted using a method reported by Kraut et al.^26–29^, i.e.

8 $${\text{VBO}}, \Delta {{\text{E}}}_{{\text{v}}}^{{\text{Ge}}-{\text{oxide}}/{\text{Ge}}(001)}=\left({BE}_{Bulk, Ge3d5/2}^{{\text{Ge}}(001)}-{E}_{VBmax}^{{\text{Ge}}(001)}\right)-\left({BE}_{Bulk, Ge3d5/2}^{Ge-oxide}-{E}_{VBmax}^{Ge-oxide}\right)-\left({BE}_{Ge(001), Ge3d5/2}^{{\text{Ge}}-{\text{oxide}}/{\text{Ge}}(001)}-{BE}_{{\text{Ge}}-{\text{oxide}},Ge3d5/2}^{{\text{Ge}}-{\text{oxide}}/{\text{Ge}}(001)}\right)$$

where ${BE}_{Bulk, Ge3d5/2}^{{\text{Ge}}(001)}$ and ${BE}_{Bulk, Ge3d5/2}^{Ge-oxide}$ are the Ge3d_5/2_ core-level peak BE of Ge(001) substrate for a clean unoxidized Ge(001) and a sufficiently oxidized Ge(001) respectively; ${E}_{VBmax}^{{\text{Ge}}(001)}$ and ${E}_{VBmax}^{Ge-oxide}$ are the valence band maxima (VB_max_) of a clean unoxidized Ge(001) and a sufficiently oxidized Ge(001) respectively; ${BE}_{Ge(001), Ge3d5/2}^{{\text{Ge}}-{\text{oxide}}/{\text{Ge}}(001)}$ is the Ge3d_5/2_ core-level BE of the Ge(001) for a partially oxidized Ge(001) while ${BE}_{{\text{Ge}}-{\text{oxide}},Ge3d5/2}^{{\text{Ge}}-{\text{oxide}}/{\text{Ge}}(001)}$ is its Ge3d_5/2_ core-level BE of Ge-oxide. The CBO can be extracted given the band gaps bulk Ge (${{\text{E}}}_{{\text{g}},{\text{bulk}}}^{{\text{Ge}}(001)}$ ≈ 0.67 eV) and bulk Ge-oxide (${{\text{E}}}_{{\text{g}},{\text{bulk}}}^{Ge-oxide}$ ≈ 6.0 eV^19^), i.e.

9 $${\text{CBO}}, \Delta {{\text{E}}}_{{\text{c}},{\text{bulk}}}^{{\text{Ge}}-{\text{oxide}}/{\text{Ge}}(001)}={{\text{E}}}_{{\text{g}},{\text{bulk}}}^{Ge-oxide}-{{\text{E}}}_{{\text{g}},{\text{bulk}}}^{{\text{Ge}}(001)}-\Delta {{\text{E}}}_{{\text{v}},{\text{bulk}}}^{{\text{Ge}}-{\text{oxide}}/{\text{Ge}}(001)}$$

**Figure 6:**
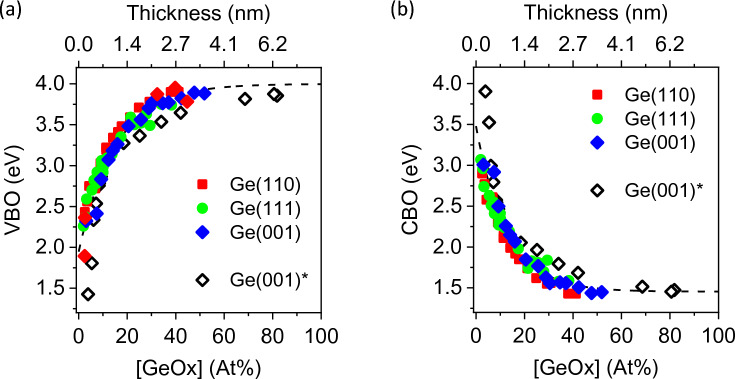
(**a**) Valence band offsets VBO and (**b**) conduction band offsets CBO plotted as a function of concentration of Ge-oxide (based on Ge3d_5/2_ BE values). VBO and CBO values do not appear to differ significantly from different Ge substrates and can therefore be treated as a universal plot. *Data extracted from Ong et al. Appl. Surf. Sci. 530 (2020) 147256.

**Figure 7:**
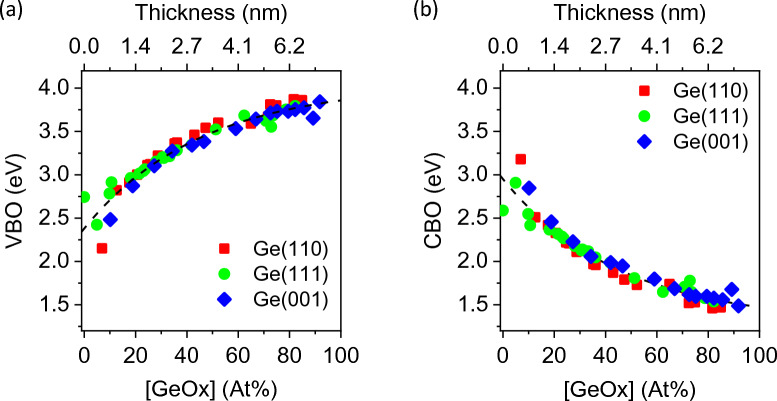
(**a**) Valence band offsets VBO and (**b**) conduction band offsets CBO plotted as a function of concentration of Ge-oxide (based on Ge2p_3/2_ BE values). The VBO and CBO values do not appear to differ significantly from different Ge substrates and can therefore be treated as a universal plot.

Figure 6 shows the values of (a) VBO and (b) CBO for Ge(001), Ge(110) and Ge(111) which are plotted as a function of time. The VBO and CBO are extracted using Ge3d core-level BE values. For consistency, the BE values of Ge2p_3/2_ are also used to extract the VBO and CBO values which are also plotted as a function of time as shown in Fig. 7. In both figures, the VBO and CBO values do not differ significantly from different Ge surfaces. Rather, it is observed that the VBO (CBO) increases (decreases) from 1.8 eV (3.5 eV) to 3.9 eV (1.5 eV) with increasing Ge-oxide concentration, *regardless of the type of Ge low-index surface*. The values are also similar in value regardless of whether Ge3d or Ge2p_3/2_ BE values are used.

**Figure 8:**
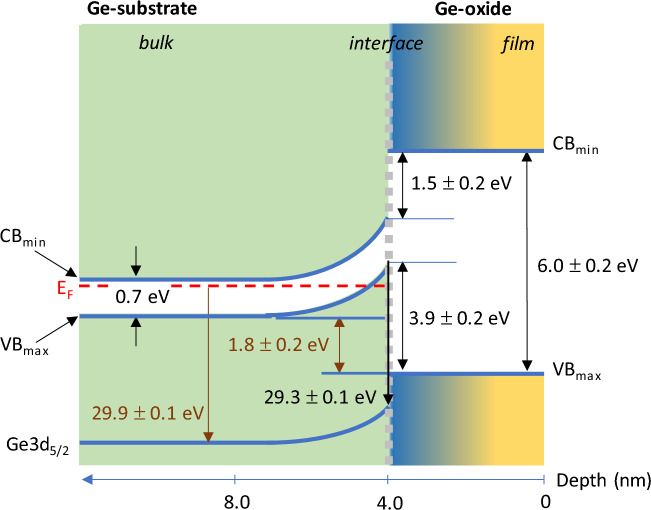
Schematic illustration depicting the band bending and band alignment of Ge/Ge-oxide. Regardless of the Ge orientation, VBO (CBO) of Ge/Ge-oxide heterostructure junction increases (decreases) from 1.8 ± 0.2 eV (3.5 ± 0.2 eV) to 3.9 ± 0.2 eV (1.5 ± 0.2 eV) when thickness is above 4 nm.

The changes in the offsets in Ge can be attributed because of transfer of charge from the bulk Ge near the surface towards the oxide overlayer during oxidation. Upon exposure to air, the initially and relatively unoxidized Ge surface oxidizes in ambient forming a thin oxide layer. XPS results suggest the formation of a sub-oxide GeO_x_ (where x < 2) at the Ge-oxide/Ge interface associated with “Ge^1+^, Ge^2+^ and Ge^3+^ species where charged carrier-trapped states are likely formed due to the presence of Ge containing varying amount of uncompensated dangling bonds in the GeO_x_ environment according to Liu et al.^18^. Their work on the structural and electronic properties of Ge/a-GeO_2_ (amorphous-GeO_2_) interface is based on density functional theory (DFT) calculations combined with consideration of sub-stoichiometric Ge (Ge^1+^, Ge^2+^ and Ge^3+^) species having different dangling-bond configurations at the interface. As oxidation proceeds, the density of charged carrier-trapped states increases as more sub-Ge species in the GeO_x_ layer are formed. Consequently, the increase in charge transfer at the interface for the n-type Ge substrate drains the electrons in Ge resulting in a larger upward band bending with depletion layer (Fig. 8). To summarize, we show that shifts in VBO and CBO values depend on the extent of Ge oxidation on the surface, not on the extent of oxidation time nor on the orientation of Ge surface. Hence, the values of VBO and CBO can be estimated for any Ge-oxide/Ge surfaces with a known oxide-concentration from Figs. 6 and 7. Note that VBO values of oxide/Ge heterostructure interfaces such as HfO_2_ deposited onto Ge(001), Ge(110) and Ge(111) layers have been reported to show different band offset values^30^. While the difference in the offset values ranges from 2.25 to 2.8 eV, the difference may also be affected by the presence of an intermediate Ge-oxide layer between HfO_2_ and Ge which was observed for 1 nm HfO_2_ films grown on Ge(001), Ge(110) and Ge(111) layers.

**Figure 9:**
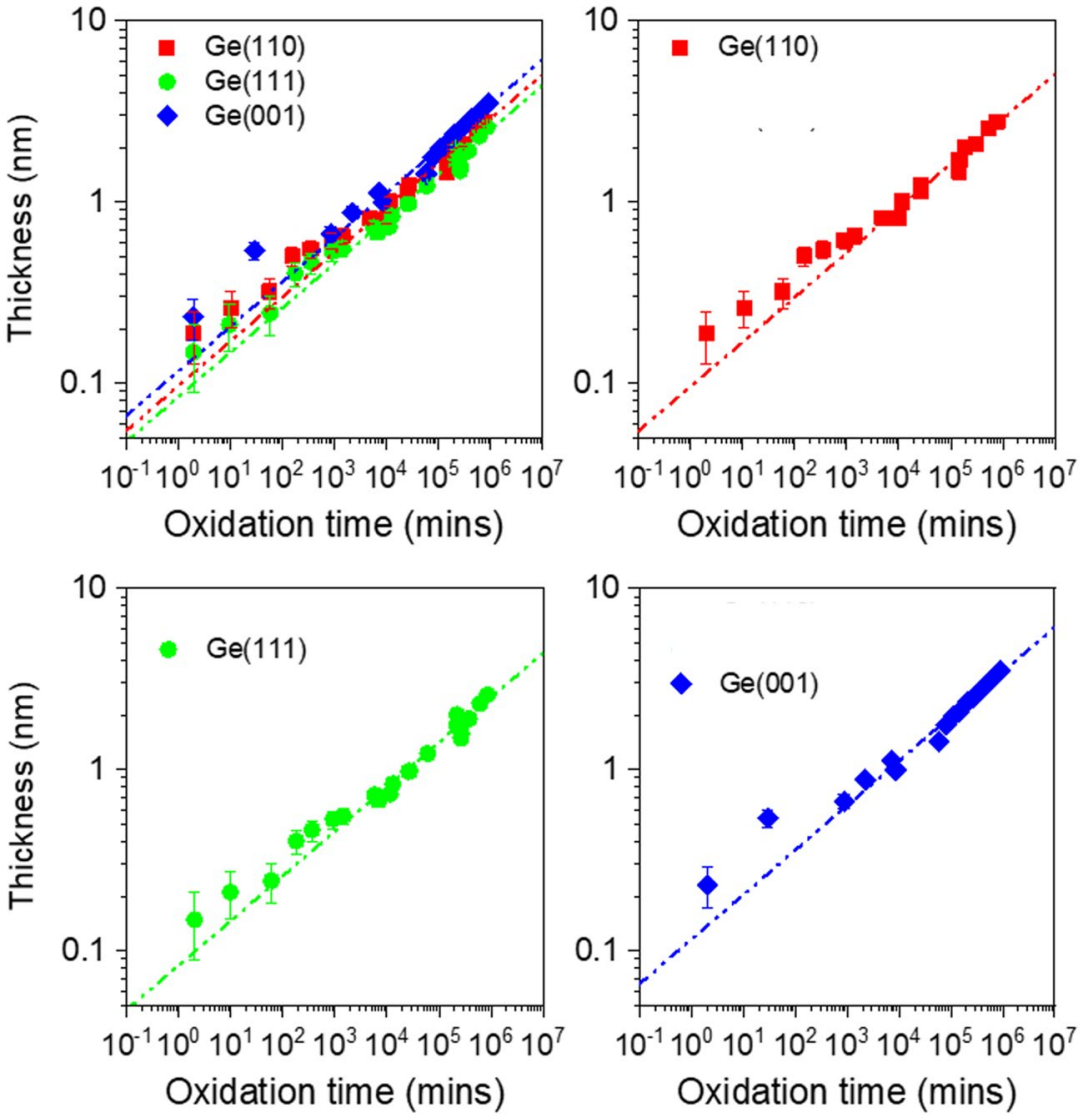
Estimated thickness of Ge-oxide which increases with oxidation time. The oxide thickness for Ge(001) is relatively higher compared to Ge(110) and Ge(111) at any time *t*. The oxidation rate (which is the slope of the trend-lines in this Figure), however, appears to be similar across the 3 substrate-orientations. For clarity, the plots are also shown individually for each surface orientation.

**Figure 10:**
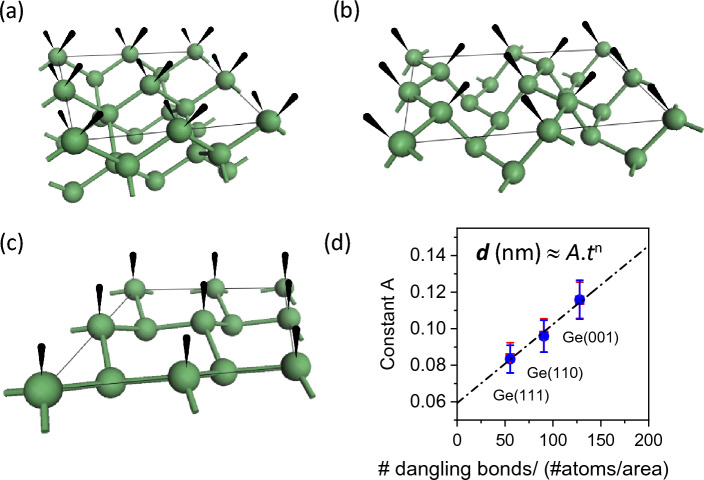
Simple models and schematic figures showing dangling bonds on the surface of (**a**) Ge(001) (**b**) Ge(110) and (**c**) Ge(111), correlating with the oxidation rates of Ge with Ge(001) being the highest, followed by Ge(110) and then Ge(111). In (**d**), the fitting parameter A increases linearly with the number of dangling bonds per Ge atom per unit area.

The thickness *d* of the oxide can also be estimated by taking the ratio of ${I}_{Ge-oxide}{/I}_{Ge3d5/2}$ using Eqs. (3) and (4), i.e.

10 $$d \left(nm\right)= \lambda .cos\theta .ln\left(\frac{{I}_{Ge-oxide}{I}_{Ge3d5/2}^{\infty }}{{I}_{Ge3d5/2}{I}_{Ge-oxide}^{\infty }}+1\right)$$

where *d* is the thickness of Ge-oxide in nm; ${I}_{Ge-oxide}$ and ${I}_{Ge3d5/2}$ are respectively the intensities of the oxide and substrate Ge3d core-levels of a partially oxidized Ge(001) sample; ${I}_{Ge3d5/2}^{\infty }$ and ${I}_{Ge-oxide}^{\infty }$ are the core-level intensities of clean (unoxidized) and fully oxidized Ge(001) samples respectively; *θ* is the photoelectron take-off angle from the sample surface normal^31^. Given that the values of λ_Ge3d5/2_ (2.342 nm) and λ_Ge-oxide_ (2.338 nm) are similar, they can be further simplified as λ (2.34 nm). The thickness of the Ge-oxide *d* (nm) is then plotted as a function of oxidation time *t* (mins) as shown in Fig. 9. The increase in the oxide thickness *d* appears to show power law dependence with oxidation time *t* given by *d* = *A.t*^*n*^ (Eq. 5) where *n ≈* 0.25, which agrees within error bar of the values reported by Ong et al.^19^. The fitted values of *n* and *A* using Eq. (5) (for Fig. 9) are also shown in Table 1b. Oxidation of Ge surfaces appear to show similar values of *n* but different values of *A* for different surface orientations. Interestingly, the values of *A* are found to be largest for Ge(001), followed by Ge(110) and lastly, Ge(111). Upon further examination, we found that the values of *A* correlate well with the number of dangling bonds per Ge atom per surface area for each Ge surface. As shown schematically in Fig. 10a–c, the values of *A* increase linearly with increasing number of dangling bonds per Ge atom per surface area (Fig. 10d). This apparent correlation suggests that the oxidation of Ge depends on the surface area density of dangling bonds on the Ge surface. Hence, for Ge(111) which contains the least number of dangling bonds per Ge atom per surface area, the oxidation rate for Ge(111) is the also the lowest compared to Ge(110) and Ge(111). Ge(001), on the other hand, has the highest number of dangling bonds per Ge atom per surface area and its oxidation appears also to be the fastest. With 2 dangling bonds per Ge atom, Ge(001) will be more susceptible to oxidation when exposed to ambient air.

## Conclusions

We examined the extend of Ge oxidation for three low-index Ge surfaces (001), (110) and (111) when exposed to ambient environment for over 500 days. Upon exposure to air, Ge-oxide (GeO_x_) grows in a pseudo layer-by-layer mode on Ge surface which increases in thickness with time. BE shifts in the Ge-oxide of Ge3d and Ge2p_3/2_ core-levels in the initial oxidation reveal Ge-oxide comprising more of Ge^1+^ and Ge^2+^ species while oxidation after an extended period of time shows more Ge^3+^ and Ge^4+^ species. On the other hand, BE shifts in Ge3d and Ge2p_3/2_ core-levels of all Ge surfaces correlate directly to the GeO_x_ (for x $\le$ 2) composition and oxide thickness, and not on the time of oxidation. All three types of Ge surfaces show a gradual decrease in BE of Ge3d (Ge2p_3/2_) core-levels from about 30.0 eV (1218.4 eV) to about 29.5 eV (1218.0 eV) with increasing oxide concentrations. A universal scaling plot for VBO (CBO) regardless of the Ge surface can thus be established, where the VBO (CBO) appears to increase (decrease) from 1.8 eV (3.5 eV) to 3.9 eV (1.5 eV) with increasing oxide concentration (thickness). Changes in the band offsets can be attributed to the presence of chemical states at the GeO_x_/Ge junction formed during oxidation. Our results show the importance of native oxide formation at the oxide–semiconductor interface and its band bending effects that can significantly affect the performance of electronic devices. The universal scaling provides an insight on how the presence of this native oxide introduces unwanted, parasitic and additional band offsets affecting the electronic properties of any oxide-germanium based heterojunctions. Interestingly, the oxidation behaviour of Ge surfaces exposed to ambient air at room temperature appears to follow an empirical power-law, where the oxide thickness *d* = *A*.t^n^ (n ≈ 0.25) and *A* increases linearly with increasing number of dangling bonds per Ge atom per surface area.

## Experimental procedures

### Sample preparation

The Ge(001), Ge(110) and Ge(111) samples were obtained by cleaving 2-inch n-type singular wafers (AXT Inc, China). The cleaved samples were then submerged in 100 ml of deionized H_2_O (Siemens Labostar Ultrapure, Resistivity 18.2 MΩ cm^−1^) for 10 min to remove the water-soluble native oxide from the wafer surface. The samples were subsequently blown dry using dry N_2_ gas and exposed to air (at room temperature of 24 ± 2 °C and relative humidity of 60 ± 5%) for time *t* (2 min up to *t* over 500 days) before being loaded into the X-Ray Photoelectron Spectroscopy (XPS) Ultra-high Vacuum (UHV) system with a base pressure of 5 × 10^–9^ mbar.

### X-Ray photoelectron spectroscopy

The XPS spectra were obtained using the Omicron EIS XPS system equipped with a monochromatic Al K_α_ X-ray source (photon energy ≈ 1486.7 eV). The binding energy (BE) of the system was calibrated using standard silver (Ag) and copper (Cu) samples where Ag3d_5/2_ and Cu3p_3/2_ peaks are located at binding energies (BE) of 368.15 eV and 932.65 eV. The incident X-ray (diameter < 1 mm spot-size) and photoelectrons were collected at 15° take-off angle with respect to the surface normal. All spectra were charge-referenced with the adventitious C1s peak corrected to 285.0 eV. For chemical states and compositional analyses, the core-level spectra were fitted using the Thermo Avantage (Thermo-Scientific) software, with error bars of BE within 0.1 to 0.2 eV. The normalized peak area *I*_*ij*_ for each element was derived by taking the normalized peak area *A*_*ij*_ of a core-level BE peak with Shirley-type background subtraction, i.e.

11 $${I}_{ij}=\frac{{A}_{ij}}{{T}_{KE}\times K\times {L}_{ij}\left(\gamma \right)\times {\sigma }_{ij}\times {\lambda }_{KE}}$$

where *A*_*ij*_ is peak area of a photoelectron core level from element *i*. *T*_*KE*_ is the analyser transmission function, *K* is an instrumental constant that includes parameters such as the irradiated sample area, X-ray flux, and photoelectrons’ solid angle taken in by the analyser. *σ*_*ij*_ is the photoionization cross-section defined as the probability the incident x-ray photon will generate a photoelectron from orbital *j* of element *I*, and *L*_*ij*_(γ) is known as the angular asymmetry factor for orbital *j* of element *i* at angle γ between emitted ele and incident X-ray. *λ*_*KE*_ is the inelastic mean free path (IMFP). The relative composition C_*ij*_ (in at%) for each element is extracted by calculating the normalized peak area I_*ij*_ for each element with respect to the other elements of interest, i.e.

12 $${C}_{ij}\left(\text{in at.\%}\right)=\frac{{I}_{ij}}{{\sum }_{i}^{j}{I}_{ij}}\times 100$$

### Atomic force microscopy

The surface morphologies of the samples cut from Ge(001), (110) and (111) wafers were obtained using the Icon Dimension Atomic Force Microscope with ScanAsyst mode using ScanAsyst-Air probes (Bruker Inc.), where scan-sizes include 250 nm × 250 nm, 500 nm × 500 nm, and 1 μm × 1 μm. Gwyddion software (version 2.55) is used to analyse the images for surface roughness values^32^.

## Data Availability

Request for data and materials should be addressed to E.S.T. (phytokes@nus.edu.sg).
